# *δ*EF1 associates with DNMT1 and maintains DNA methylation of the E-cadherin promoter in breast cancer cells

**DOI:** 10.1002/cam4.347

**Published:** 2014-10-15

**Authors:** Akihiko Fukagawa, Hiroki Ishii, Keiji Miyazawa, Masao Saitoh

**Affiliations:** 1 Department of Biochemistry, University of Yamanashi YamanashiYamanashi, Chuo, 409-3898, Japan; 2 Research Training Program for Undergraduates, Interdisciplinary Graduate School of Medicine and Engineering, University of Yamanashi YamanashiYamanashi, Chuo, 409-3898, Japan

**Keywords:** Cancer cells, DNA methylation, E-cadherin, EMT, *δ*EF1

## Abstract

Abnormal DNA methylation at the C-5 position of cytosine (5mC) of CpG dinucleotides is a well-known epigenetic feature of cancer. Levels of E-cadherin, which is regularly expressed in epithelial tissues, are frequently reduced in epithelial tumors due to transcriptional repression, sometimes accompanied by hypermethylation of the promoter region. *δ*EF1 family proteins (*δ*EF1/ZEB1 and SIP1/ZEB2), key regulators of the epithelial-mesenchymal transition (EMT), suppress E-cadherin expression at the transcriptional level. We recently showed that levels of mRNAs encoding *δ*EF1 proteins are regulated reciprocally with E-cadherin level in breast cancer cells. Here, we examined the mechanism underlying downregulation of E-cadherin expression in three basal-type breast cancer cells in which the E-cadherin promoter region is hypermethylated (Hs578T) or moderately methylated (BT549 and MDA-MB-231). Regardless of methylation status, treatment with 5-aza-2′-deoxycytidine (5-aza), which inhibits DNA methyltransferases, had no effect on E-cadherin expression. Knockdown of *δ*EF1 and SIP1 resulted in recovery of E-cadherin expression in cells lacking hypermethylation, whereas combined treatment with 5-aza synergistically restored E-cadherin expression, especially when the E-cadherin promoter was hypermethylated. Moreover, *δ*EF1 interacted with DNA methyltransferase 1 (DNMT1) through the Smad-binding domain. Sustained knockdown of *δ*EF1 family proteins reduced the number of 5mC sites in the E-cadherin promoter region, suggesting that these proteins maintain 5mC through interaction with DNMT1 in breast cancer cells. Thus, *δ*EF1 family proteins appear to repress expression of E-cadherin during cancer progression, both directly at the transcriptional level and indirectly at the epigenetic level.

## Introduction

Breast cancer is a heterogeneous disease comprising a variety of pathologies that exhibit a wide range of histological characteristics and clinical outcomes. According to gene expression profiling, human breast cancers can be classified into at least five molecular subtypes 1. Among these, the major subtypes are luminal and basal like, originating from two distinct types of epithelial cells found in the normal mammary gland. The luminal subtype, which is generally estrogen receptor- and progesterone receptor-positive, exhibits low malignancy and a good prognosis following multiple therapeutic modalities, especially hormone therapy. The basal-like subtype exhibits mesenchymal features, metastasis-associated phenotypes, aggressive behavior, and poor prognosis. Recently, basal-like tumors have been further categorized into two subtypes, Basal A and Basal B. The Basal A subtype has a basal-like signature and is positive for basal cytokeratin (K5/K14), whereas the Basal B subtype exhibits a stem cell-like expression profile, is positive for vimentin, and may reflect the clinical triple-negative tumor type 2,3. Therefore, it is necessary to identify molecular signatures and signaling pathways that contribute to malignant phenotypes of the cells.

The process of cancer-cell invasion involves the loss of cell–cell interactions along with acquisition of motile properties, and is often associated with epithelial-mesenchymal transition (EMT) of the cells 4. Formation of tight cell–cell adhesions is mainly dependent on the E-cadherin system 5. Repression of E-cadherin, frequently observed in human malignant tumors, is mediated at the transcriptional level by the *δ*EF1 family of two-handed zinc-finger factors (*δ*EF1/ZEB1 and SIP1/ZEB2), proteins of the Snail family (Snail, Slug, and Smuc), and basic helix-loop-helix factors (Twist and E12/E47) 4. Loss of E-cadherin also may reflect mutation of the coding region of the E-cadherin gene or epigenetic modifications to the DNA in the promoter region 6.

One of the fundamental epigenetic modifications in DNA is methylation of the C-5 position of cytosine (5mC) in CpG dinucleotides. The specific transfer of methyl groups to form 5mC is catalyzed by members of the DNA methyltransferase (DNMT) protein family 7. DNMT2 and DNMT3 (which has three isoforms: DNMT3a, DNMT3b, and DNMT3L) induce de novo methylation in ummethylated CpG. On the other hand, DNMT1 preferentially methylates DNA containing hemi-methylated CpG, and is implicated in copying and maintaining methylation patterns from the parental to the daughter strand during DNA replication. Several 5mC-binding proteins have been identified; these factors are called methyl-CpG-binding domain (MBD) proteins 8. Among them, MBD2 and 3 form a complex with DNMT1 and are colocalized at hemi-methylated DNA 9.

Previously, we performed mass-spectrometry analysis to search for *δ*EF1-interacting proteins, resulting in identification of MBD2 and 3 (unpublished data). In addition, we recently reported that *δ*EF1 is highly expressed in basal-like subtype cells with low E-cadherin levels 10. In this study, we found that *δ*EF1 bound to DNMT1 in breast cancer cells of the basal-like subtype, and that silencing of *δ*EF1 family proteins (*δ*EF1/ZEB1 and SIP1/ZEB2) considerably decreased the number of 5mC sites. Together, these findings suggest that in aggressive cancer cells, *δ*EF1 recruits DNMT1 to hemi-methylated DNA in the promoter region of E-cadherin, resulting in reduced expression of E-cadherin via hypermethylation. Therefore, *δ*EF1 acts as a transcriptional repressor to directly suppress E-cadherin, and as a potent epigenetic regulator (in cooperation with DNMT1) to maintain E-cadherin repression.

## Materials and Methods

### Cell culture, antibodies, and reagents

All cells were cultured in Dulbecco's modified Eagle's medium (DMEM; Nacalai Tesque, Kyoto, Japan) supplemented with 10% fetal bovine serum (FBS), 50 U/mL penicillin, and 50 *μ*g/mL streptomycin (Nacalai Tesque). Hs578T and BT549 cells were cultured in DMEM in the presence of 10% FBS, 10 *μ*g/mL insulin, and the same antibiotics. To produce lentivirus, HEK293FT cells were cultured in DMEM supplemented with 10% FBS, 2 mmol/L l-glutamine, 0.1 mmol/L MEM nonessential amino acids (Invitrogen, Carlsbad, CA), and 1 mmol/L MEM sodium pyruvate (Invitrogen). All cells were grown in a 5% CO_2_ atmosphere at 37°C. Transient transfection of expression plasmids and siRNAs was performed using Lipofectamine 2000 and Lipofectamine RNAiMAX, respectively (Invitrogen). Mouse monoclonal anti-FLAG M2 and anti-*α*-tubulin antibodies were purchased from Sigma-Aldrich (St. Louis, MO). Mouse monoclonal anti-DNMT1 and anti-Myc antibodies were purchased from IMGENEX (San Diego, CA) and BD Biosciences (San Jose, CA), respectively. Rabbit polyclonal *δ*EF1 antibody was purchased from Novus Biologicals (Littleton, CO). Mouse anti-E-cadherin and rat anti-HA (3F10) antibodies were from BD Transduction Laboratories (Lexington, KY) and Roche Applied Science (Penzberg, Germany), respectively. The DNA methyltransferase inhibitor 5-aza-2′-deoxycytidine was obtained from Sigma-Aldrich.

### RNA extraction and quantitative RT-PCR

Total RNA was purified using Isogen (Nippon Gene, Tokyo, Japan). cDNAs were synthesized using the Prime Script First Strand cDNA Synthesis kit (TAKARA, Otsu, Japan). Quantitative (qRT-PCR) was performed using Power SYBR Green PCR Master Mix (Applied Biosystems, Foster City, CA). Values in each sample were normalized to the corresponding level of the mRNA-encoding human glyceraldehyde-3-phosphate dehydrogenase (GAPDH). PCR reactions were performed using the following primers: *δ*EF1: forward, 5′-TGCACTGAGTGTGGAAAAGC-3′, reverse, 5′-TTGCAGTTTGGGCATTCATA-3′; SIP1: forward, 5′-AAGCCCCATCACCCATACAAG-3′, reverse, 5′-AAATTCCTGAGGAAGGCCCA-3′; E-cadherin: forward, 5′-TGCACCAACCCTCATGAGTG-3′, reverse, 5′-GTCAGTATCAGCCGCTTTCAG-3′; GAPDH: forward, 5′-CGACCACTTTGTCAAGCTCA-3′, reverse, 5′-CCCTGTTGCTGTAGCCAAAT-3′.

### Bisulfite sequencing

Genomic DNA was isolated using the DNeasy Tissue Kit (Qiagen, Valencia, CA). Genomic DNA was treated with sodium bisulfite using the EZ DNA Methylation-Gold kit (Zymo Research, Irvine, CA). Bisulfite-reacted DNAs were used as templates for PCR amplification of the site including the E-boxes in the E-cadherin promoter. The primers for the E-cadherin promoter were as follows. First reaction: forward, 5′-ATTTTAGTAATTTTAGGTTAGAGGG-3′; reverse 5′-TCCAAAAACCCATAACTAACC-3′. Second reaction: forward, 5′-AGTAATTTTAGGTTAGAGGGTT-3′, reverse, 5′-CTAAAATCTAAACTAACTTC-3′. These PCR products were inserted into the TA cloning vector (Invitrogen) for sequencing.

### Plasmid constructions and RNA interference

All plasmids and siRNAs used in this study were previously described 11,12. Oligonucleotide sequences used for shRNAs against *δ*EF1 and SIP1 were as follows. *δ*EF1: top strand, 5′-CACCGCTACTGGAGATGGCAATTGCCAACAAATTGCCATCTCCAGTAGC-3′; bottom strand, 5′-AAAAGCTACTGGAGATGGCAATTTGTTCGCAAATTGCCATCTCCAGTAGC-3′. SIP1: top strand, 5′-CACCGGAGAAAGTACCAGCGGAAACCGAAGTTTCCGCTGGTACTTTCTCC-3′; bottom strand, 5′-AAAAGGAGAAAGTACCAGCGGAAACTTCGGTTTCCGCTGGTACTTTCTCC-3′. LacZ (as a negative control): top strand, 5′-CACCAAATCGCTGATTTGTGTAGTCGGAGACGACTACACAAATCAGCGA-3′; bottom strand, 5′-AAAATCGCTGATTTGTGTAGTCGTCTCCGACTACACAAATCAGCGATTT-3′. The oligonucleotides were shuttled from the pENTR/H1/TO vector into the pCS-RfA-CG vector by the Gateway technique (Invitrogen). To generate lentiviruses, HEK293FT cells were transiently transfected with plasmids encoding pCAG-HIV-gp, pCMV-VSV-G-RSV-Rev and pCS-RfA-CG using Lipofectamine 2000 (Invitrogen). Twelve hours after transfection, the culture medium was changed, and the cells were cultivated further for 72 h. The supernatant was harvested, cleared by centrifugation and filtration, and used for infection.

### Immunoprecipitation, immunoblotting, and immunofluorescence labeling

Cells were lysed in Lysis buffer solution (20 mmol/L Tris-HCl, pH 7.5, 150 mmol/L NaCl, 10 mmol/L ethylenediaminetetraacetic acid, 1 mmol/L ethyleneglycoltetraacetic acid, 1% Nonidet P-40). After measurement of protein concentrations with a BCA Protein Assay Kit (Pierce, Rockford, IL), equal amounts of total protein per lane were subjected to SDS gel-electrophoresis (SDS-PAGE), followed by semidry transfer of the proteins to Fluoro Trans W membrane (Pall, Glen Cove, NY). After clearing with centrifugation, the supernatants were incubated with the indicated antibodies for 1 h and then incubated with Protein G-Sepharose (Amersham, Piscataway, NJ) for another 30 min. After the beads were washed twice with the cell lysis buffer, proteins were subjected to SDS-PAGE. Nonspecific binding of proteins to the membrane was blocked by incubation in Tris-buffered saline-T buffer (50 mmol/L Tris-HCl, pH 7.4, 150 mmol/L NaCl, and 0.1% Tween-20) containing 5% skim milk. Immunodetection was performed with the ECL blotting system (Amersham). Cells seeded onto 8-well culture slides (BD Biosciences) were fixed with 1:1 acetone-methanol solution and washed 5 times with phosphate-buffered saline. After cells were incubated with primary antibody in Blocking One (Nacalai Tesque), they were further incubated with secondary antibodies and TOTO3 (Invitrogen Molecular Probes, Eugene, OR) for 1 h. Fluorescence was examined by confocal laser scanning microscopy.

## Results

### Reciprocal control of expression between *δ*EF1 and E-cadherin in human breast cancer cells

**Figure 1:**
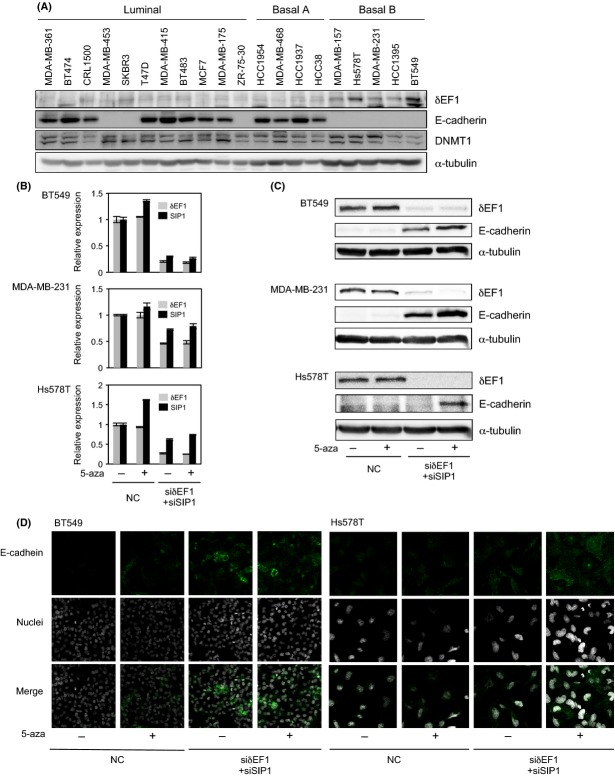
Expression profiles of E-cadherin, *δ*EF1, and DNMT1 in human breast cancer cells. (A) Protein levels of E-cadherin, *δ*EF1, and DNMT1 were determined by immunoblot analysis of whole-cell extracts. *α*-tubulin levels were monitored as a loading control. Molecular subtypes are as reported by Neve et al. 2. and Charafe-Jauffret et al. 3. (B, C, and D) BT549, MDA-MB-231, and Hs578T cells were transfected with siRNAs against both *δ*EF1 and SIP1, and then treated with 5 *μ*mol/L of 5-aza-2′-deoxycytidine (5-aza) for 48 h (for BT549 and MDA-MB-231 cells) or 1 *μ*mol/L of 5-aza for 72 h (for Hs587T cells). Cells were then harvested and examined for expression of *δ*EF1, SIP1, and E-cadherin by quantitative RT-PCR (B), immunoblotting (C), or immunocytochemistry (D). NC, negative control.

We previously reported that expression of *δ*EF1 at the mRNA level is inversely correlated with that of E-cadherin 10. To confirm this finding at the protein level, we detected both factors by immunoblotting samples from human breast cancer cell lines. As with the mRNA levels, the protein levels of *δ*EF1 and E-cadherin were reciprocally regulated: most cell lines with low *δ*EF1 levels and high E-cadherin levels were categorized into the luminal and Basal A subtypes of breast cancer, whereas those with high *δ*EF1 levels and low E-cadherin levels were categorized into the Basal B subtype (Fig.1A). *δ*EF1 and SIP1 associate with common E-box sequences and function redundantly 10,13. Therefore, we examined E-cadherin levels by qRT-PCR and immunoblotting after simultaneously knocking down *δ*EF1 and SIP1 in BT549 and MDA-MB-231 cells, as well as Hs578T cells. Transfection of both siRNAs resulted in moderate upregulation of E-cadherin in BT549 and MDA-MB-231 cells, but little upregulation in Hs578T cells (Fig.1B and C). Addition of 5-aza-2′-deoxycytidine (5-aza) alone, an inhibitor of DNA methyltransferases, was not sufficient to restore E-cadherin expression in these cells. However, this compound enhanced the effects of the siRNAs on recovery of E-cadherin expression, especially in Hs578T cells (Fig.1B and C). These findings were also confirmed by immunocytochemical analyses (Fig.1D), suggesting that E-cadherin is maintained at low levels by both *δ*EF1/SIP1 and a DNA methyltransferase.

### DNA hypermethylation in the promoter region of E-cadherin

**Figure 2:**
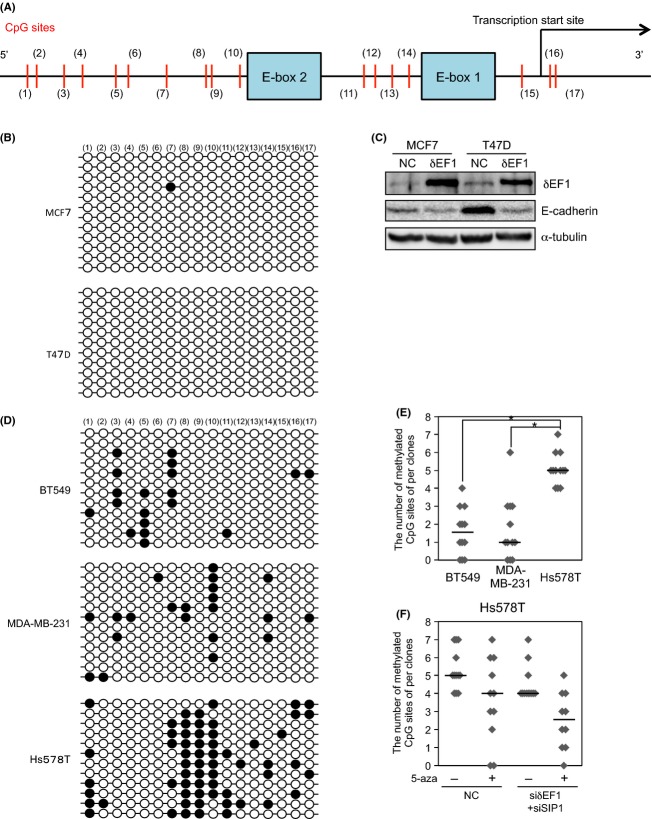
DNA methylation at the C-5 position (5mC) of cytosine in CpG dinucleotides in human breast cancer cells. (A) Schematic illustration of the promoter region of human E-cadherin (−162 to +37). Numbers in parentheses represent individual CpG sites in the region. E-boxes 1 and 2 have been already reported as binding sites for *δ*EF1 and SIP1^13^–^15^. (B) Bisulfite sequencing was performed using bisulfite-treated templates from MCF7 and T47D cells. White and black circles represent unmethylated and methylated CpG (5mC) sites, respectively. (C) MCF7 and T47D cells were infected with lentiviral vectors encoding FLAG-*δ*EF1. After 48 h, immunoblots were performed on whole-cell extracts. *α*-tubulin levels were monitored as a loading control. (D) Bisulfite sequencing was performed using bisulfite-treated templates from BT549, MDA-MB-231, and Hs578T cells. White and black circles represent unmethylated and methylated CpG (5mC), respectively. (E) The number of 5mC sites was compared among BT549, MDA-MB-231, and Hs578T cells. The Mann–Whitney *U*-test was used for assessing distributional differences of variance across different test samples. *Mann–Whitney *U*-test, *P* < 0.01. (F) Hs578T cells transfected with siRNAs against both *δ*EF1 and SIP1 were treated with 1 *μ*mol/L 5-aza-2′-deoxycytidine (5-aza) for 72 h. After bisulfite sequencing was performed on 11 clones of Hs578T treated with the indicated combinations, the number of methylated CpG (5mC) sites was counted. Median values are represented by horizontal bars (E and F). NC, negative control.

DNA hypermethylation in the promoter region of E-cadherin represses E-cadherin expression in various types of cancer cells 6. Although addition of 5-aza alone did not upregulate E-cadherin in the cells used in this study, 5-aza in combination with the siRNAs synergistically increased the expression of E-cadherin. To elucidate the underlying mechanism, we used bisulfite sequencing to examine DNA methylation status of the E-cadherin promoter region in breast cancer cells. Because *δ*EF1 and SIP1 can associate with two E-box sites in the promoter region of E-cadherin 13–15, we focused on the region adjacent to these two E-boxes (Fig.2A). MCF7 and T47D cells, representatives of the luminal subtype, exhibited only a few 5mC sites in the region, consistent with their high levels of E-cadherin expression (Figs.1A and2B). In addition, overexpression of *δ*EF1 reduced E-cadherin expression without affecting methylation status (Figs.2C and S1A). Conversely, BT549 and MDA-MB-231 cells exhibited a moderate number of DNA methylations in this region (Fig.2D and E). E-cadherin expression was partially restored following treatment with both *δ*EF1 and SIP1 siRNAs, but this elevation in E-cadherin level was not accompanied by a reduction in the number of 5mC sites (Fig. S1B and see Fig.1C). These findings indicate that *δ*EF1/SIP1 directly regulate E-cadherin expression at the transcriptional level in cells without hypermethylation in the E-cadherin promoter region. However, Hs578T cells exhibited hypermethylation in the region relative to the other cell types we examined (Fig.2D and E). Treatment of these cells with either 5-aza or the siRNAs alone marginally decreased the number of 5mC sites, but did not upregulate E-cadherin expression (Fig.2F and see Fig.1C). Furthermore, methylation status was decreased (although not significantly) by combined treatment with 5-aza and siRNAs, resulting in E-cadherin upregulation (Figs.2F and S1C, and see Fig.1C). Together, these findings suggest that hypermethylation in the E-cadherin promoter region is maintained by *δ*EF1/SIP1 and a DNA methyltransferase in Hs578T cells.

### Interaction of *δ*EF1 with DNA methyltransferase

**Figure 3:**
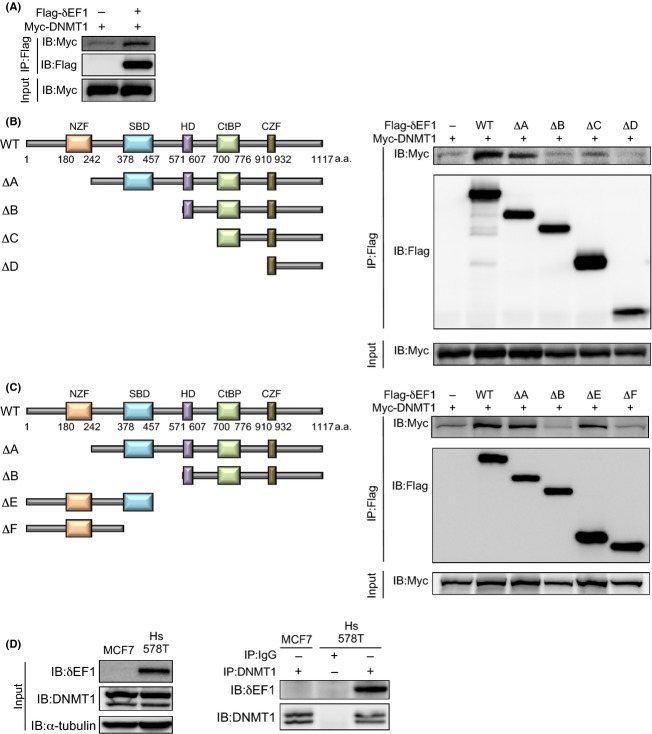
Interaction of *δ*EF1 with DNMT1. (A–C) HEK293 cells were transiently transfected with the indicated expression plasmids. Twenty-four hours after transfection, cells were harvested, lysed, and subjected to immunoprecipitation (IP) with anti-FLAG antibody, followed by immunoblotting (IB) with anti-Myc antibody. Schematic illustrations depict wild-type (WT), N-terminally truncated mutants (ΔA–ΔD), and C-terminally truncated mutants (ΔE–ΔF) of *δ*EF1 (left panels in B and C). (D) MCF7 and Hs578T cells were harvested and subjected to immunoprecipitation (IP) with anti-DNMT1 antibody or IgG, followed by immunoblotting (IB) with anti-DNMT1 or anti-*δ*EF1 antibodies. *α*-tubulin levels were monitored as a loading control. NZF, N-terminal zinc finger; SBD, Smad-binding domain; HD, homeodomain; CtBP, CtBP-binding domain; CZF, C-terminal zinc finger.

Previously, we performed mass-spectrometry analysis to determine which proteins bind to *δ*EF1, and identified MBD2 and 3 as *δ*EF1-binding proteins (unpubl. data). MBD2 and 3 bind to hemi-methylated DNA and form a complex with DNMT1 9. Therefore, we investigated whether *δ*EF1 interacted with DNMT1 in HEK293 cells ectopically overexpressing FLAG-*δ*EF1 and Myc-DNMT1. FLAG-*δ*EF1 interacted with Myc-DNMT1, as well as HA-MBD2 and HA-MBD3 (Figs.3A and S2A). Furthermore, FLAG-*δ*EF1 lacking the N-terminal zinc-finger (NZF) domain (mutant ΔA) also interacted with DNMT1, whereas FLAG-*δ*EF1 lacking the Smad-binding domain (SBD) (mutants ΔB–ΔD) did not (Fig.3B). Moreover, an N-terminal mutant of *δ*EF1 containing the SBD (mutant ΔE) also interacted with DNMT1 (Fig.3C), suggesting that *δ*EF1 interacted with DNMT1 through its SBD. However, although Smads reportedly interact with *δ*EF1 in a TGF-*β*-dependent manner, binding of *δ*EF1 to DNMT1 was not affected by TGF-*β* stimulation (data not shown). Next, we confirmed this interaction in Hs578T cells. Figure3D shows that endogenous *δ*EF1 coimmunoprecipitated with DNMT1 in Hs578T cells, but not MCF7 cells, in which DNMT1 was expressed at levels similar to those in cells of the basal-like subtype (see Fig.1A). These findings suggest that *δ*EF1 constitutively interacts with DNMT1 at hemi-methylated DNA of the E-cadherin promoter region, where it is likely to be responsible for copying and maintaining DNA methylation, resulting in severe repression of E-cadherin expression in Hs578T cells.

### Reduced levels of 5mC in the E-cadherin promoter region in cells stably expressing shRNAs against both *δ*EF1 and SIP1

**Figure 4:**
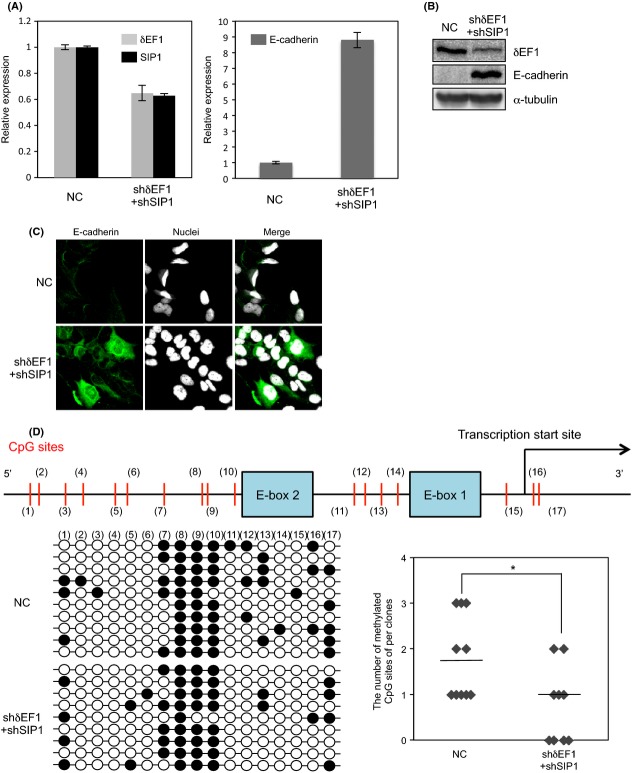
Evaluation of 5mC sites after sustained knockdown of both *δ*EF1 and SIP1 in Hs578T. (A, B, and C) Lentiviral vectors encoding *δ*EF1 and SIP1 shRNAs were used to infect Hs578T cells. Twenty days after infection, the cells were harvested and examined for expression of *δ*EF1/SIP1 and E-cadherin by quantitative RT-PCR (A), immunoblotting (B), or immunofluorescence (C). (D) Schematic illustration of the promoter region of human E-cadherin is shown (top). After bisulfite sequencing was performed, the number of methylated CpG (5mC) sites ay (11)–(17) was counted (right). White and black circles represent unmethylated and methylated CpG (5mC) sites, respectively (left). Median values are represented as horizontal bars (right). NC, negative control.

Because *δ*EF1 interacted with DNMT1, we next investigated whether *δ*EF1 is involved in copying methylation at the E-cadherin locus onto the newly synthesized strand, in cooperation with DNMT1. To assess this, because DNMT1 is implicated in the “maintenance methylation” of the nascent DNA strand after replication of methylated DNA 16, we simultaneously silenced *δ*EF1 and SIP1 in Hs578T cells over a long period of time. Twenty days after Hs578T cells were infected with lentiviral vectors encoding shRNAs against *δ*EF1 and SIP1, the cells exhibited an approximately 40% reduction in the expression levels of endogenous *δ*EF1 and SIP1, along with dramatic upregulation of E-cadherin expression, as determined by qPCR, immunoblotting, and immunocytochemical analyses (Fig.4A, B, and C). The number of 5mC sites was slightly but significantly decreased in the region adjacent to E-box1, but not in E-box2, even in the absence of 5-aza (Fig.4D, compared to Fig.2F). Although overexpression of *δ*EF1 alone downregulated E-cadherin expression, it was not sufficient to increase the number of 5mC sites in BT549 and MDA-MB-231 cells (Fig. S3 and data not shown). Thus, the number of 5mC sites was affected only by long-term knockdown of both *δ*EF1 and SIP1, suggesting that *δ*EF1/SIP1 are necessary, but not sufficient, to maintain 5mC sites in the E-cadherin promoter region.

In conclusion, *δ*EF1 may associate with DNMT1, as well as MBD2 and 3, to establish and/or maintain methylation patterns in the E-cadherin promoter region in cancer cells. Together with previously published observations, our results demonstrate that *δ*EF1 acts as a transcriptional repressor as well as an epigenetic regulator of E-cadherin during EMT and cancer progression.

## Discussion

Expression levels of E-cadherin and *δ*EF1 are reciprocally regulated in breast cancer cells. Recently, we reported that epithelial splicing regulatory proteins (ESRPs) are also transcriptionally suppressed by *δ*EF1/SIP1 during the EMT and in cells of the basal-like subtype 10. Similar to the recovery of E-cadherin observed in BT549 and MDA-MB-231 cells, knockdown of *δ*EF1 and SIP1 modestly upregulates ESRPs even in Hs578T cells. Treatment with 5-aza alone, however, does not significantly induce re-expression of ESRPs. In the cells used in this study, we detected no synergistic effects of 5-aza and *δ*EF1/SIP1 siRNAs on the expression of ESRPs (data not shown). Thus, ESRPs were repressed mainly at the transcriptional level by *δ*EF1/SIP1, whereas E-cadherin, at least in Hs578T cells, was synergistically regulated by *δ*EF1/SIP1 and DNA methylation. Therefore, it is likely that E-cadherin is directly repressed at the transcriptional levels by *δ*EF1/SIP1, as well as indirectly regulated at the epigenetic level, by *δ*EF1/SIP1 in collaboration with DNMTs.

We recently reported that association of *δ*EF1 with the promoter region of ESRP genes can be clearly detected in a chromatin immunoprecipitation (ChIP) assay 10, probably because *δ*EF1 binds directly to this region to regulate transcription. However, in ChIP assays performed in Hs578T cells, we detected no association of *δ*EF1 with methylated DNA in the E-cadherin promoter region (data not shown). These findings suggested that *δ*EF1 indirectly interacts with 5mC sites with low affinity by forming an intricate molecular complex. Indeed, MBDs directly bind to 5mC sites and are components of the nucleosome remodeling and deacetylase (NuRD) complex. The NuRD complex, which modulates transcription by influencing the status of chromatin remodeling, contains six subunits other than MBD3 (or MBD2): the histone deacetylase core proteins (HDAC), the histone-binding proteins, the metastasis-associated proteins (MTA1–3), and the chromodomain/helicase/DNA-binding protein CHD3 (or CHD4) 17. Because *δ*EF1 interacted with MBD2 and 3, we investigated whether *δ*EF1 could associate with other components of NuRD complex. We found that MTA1 and 2 interacted with *δ*EF1 (Fig. S2C and D), suggesting that *δ*EF1 could be involved in the NuRD complex. Thus, it appears that MBD2 and 3, which associate directly with 5mC sites, interact with *δ*EF1 and recruit DNMT1 and MTAs to form the NuRD complex at hemi-methylated DNA in the E-cadherin promoter region.

Epigenetic regulation of gene expressions is hierarchically regulated by covalent modifications of histone, which preceded methylation of promoter DNA. In the present study, overexpression of *δ*EF1 alone failed to induce the DNA methylation of E-cadherin promoter (Fig. S3B), suggesting that it is not sufficient to alter histone modification and chromatin structure. So far, chromatin-modifying proteins, which interact with *δ*EF1, are not identified, while Snail, another key regulator of EMT, interacts with DNMT1 as well as multiple chromatin-modifying proteins including LSD1 (histone lysine-specific demethylase), PRC2 (Polycomb repressive complex 2), and Suv39H1 (histone methyltransferase responsible for the trimethylation of H3K9) [18, 19]. Thus, it seems that *δ*EF1 preferentially controls DNA methylation of E-cadherin promoter through forming NuRD complex, whereas Snail regulates both methylation status and chromatin modification of E-cadherin gene, albeit both *δ*EF1 and Snail also act as transcriptional repressors.

Cancer cells that have acquired invasive properties frequently express lower levels of E-cadherin. Thus, this reduction in expression is a hallmark of the EMT, and seems to be transcriptionally regulated, because cancer cells regain epithelial properties at sites of distant metastasis by a reverse process called the mesenchymal-epithelial transition (MET) 4. However, numerous reports have demonstrated the presence of hypermethylation in the E-cadherin promoter region in cancer cells with aggressive phenotypes, including high invasive capacities. Because DNMTs catalyze the formation of 5mC at CpG sites, our results raise the possibility that *δ*EF1 functions to recruit DNMT1, along with the NuRD complex, to promoter regions where *δ*EF1 is already present and acting as a transcriptional repressor. However, it is still unclear how *δ*EF1 exchanges the transcriptional repression complex to the NuRD complex to regulate the methylation status of the E-cadherin promoter. Based on the results of this study and others, we propose that suppression of *δ*EF1 function represents a promising strategy for treatment of breast cancer progression.

## Supporting Information

**Figure S1.** Effect of overexpression of *δ*EF1 on methylated CpG (5mC) sites in human breast cancer cells. (A) Schematic illustration of the promoter region of human E-cadherin (top). Lentiviral vector encoding *δ*EF1 was used to infect MCF7 and T47D cells. Two days later, the number of methylated CpG (5mC) sites was determined bisulfite sequencing. White and black circles represent unmethylated and methylated CpG (5mC), respectively. (B) BT549 and MDA-MB-231 cells were transiently transfected with siRNAs against both *δ*EF1 and SIP1. Two days later, the number of methylated CpG (5mC) sites was determined bisulfite sequencing. Mean values are represented as horizontal bars (right). NC, negative control. (C) After combined treatment with 5-aza and siRNAs, bisulfite sequencing was performed. White and black circles represent unmethylated and methylated CpG (5mC) sites, respectively. **Figure S2.** Interaction of *δ*EF1 with the NuRD complex proteins, MBD2, MBD3, MTA1, and MTA2. (A, B, C, and D) HEK293 cells were transiently transfected with the indicated plasmids. Twenty-four hours after transfection, cells were harvested, lysed, and subjected to immunoprecipitation (IP) with anti-FLAG antibody, followed by immunoblotting (IB) with anti-FLAG, anti-Myc, and anti-HA antibodies. **Figure S3.** Effects of long-term overexpression of *δ*EF1 on methylated CpG (5mC) sites in BT 549 cells. (A and B) Twenty days after lentiviral vector encoding *δ*EF1 was used to infect BT549 cells, the cells were examined for expression of E-cadherin by qRT-PCR analysis (A), and for the number of methylated CpG (5mC) sites by bisulfite sequencing (B). White and black circles represent unmethylated and methylated CpG (5mC), respectively (B). NC, negative control.
