# Supplementary material for: δEF1 associates with DNMT1 and maintains DNA methylation of the E-cadherin promoter in breast cancer cells
Source: Cancer Med. 2014 Oct 15;4(1):125–35. doi: 10.1002/cam4.347 (PMC4312126; doi:10.1002/cam4.347)

Supporting information

Supplemental Figure S1 Effect of overexpression of δEF1 on methylated CpG (5mC) sites in human breast cancer cells. (**A**) Schematic illustration of the promoter region of human E-cadherin (top). Lentiviral vector encoding δEF1 was used to infect MCF7 and T47D cells. Two days later, the number of methylated CpG (5mC) sites was determined bisulfite sequencing. White and black circles represent unmethylated and methylated CpG (5mC), respectively. (**B**) BT549 and MDA-MB-231 cells were transiently transfected with siRNAs against both δEF1 and SIP1. Two days later, the number of methylated CpG (5mC) sites was determined bisulfite sequencing. Mean values are represented as horizontal bars (right). NC, negative control. (**C**) After combined treatment with 5-aza and siRNAs, bisulfite sequencing was performed. White and black circles represent unmethylated and methylated CpG (5mC) sites, respectively.

Supplemental Figure S2 Interaction of δEF1 with the NuRD complex proteins, MBD2, MBD3, MTA1, and MTA2. (**A, B, C, and D**) HEK293 cells were transiently transfected with the indicated plasmids. Twenty-four hours after transfection, cells were harvested, lysed, and subjected to immunoprecipitation (IP) with anti-FLAG antibody, followed by immunoblotting (IB) with anti-FLAG, anti-Myc, and anti-HA antibodies.

Supplemental Figure S3 Effects of long-term overexpression of δEF1 on methylated CpG (5mC) sites in BT 549 cells**.** **(A and B)** Twenty days after lentiviral vector encoding δEF1 was used to infect BT549 cells, the cells were examined for expression of E-cadherin by qRT-PCR analysis (A), and for the number of methylated CpG (5mC) sites by bisulfite sequencing (B).

White and black circles represent unmethylated and methylated CpG (5mC), respectively (B).

NC, negative control.

Supplemental figure S1

A

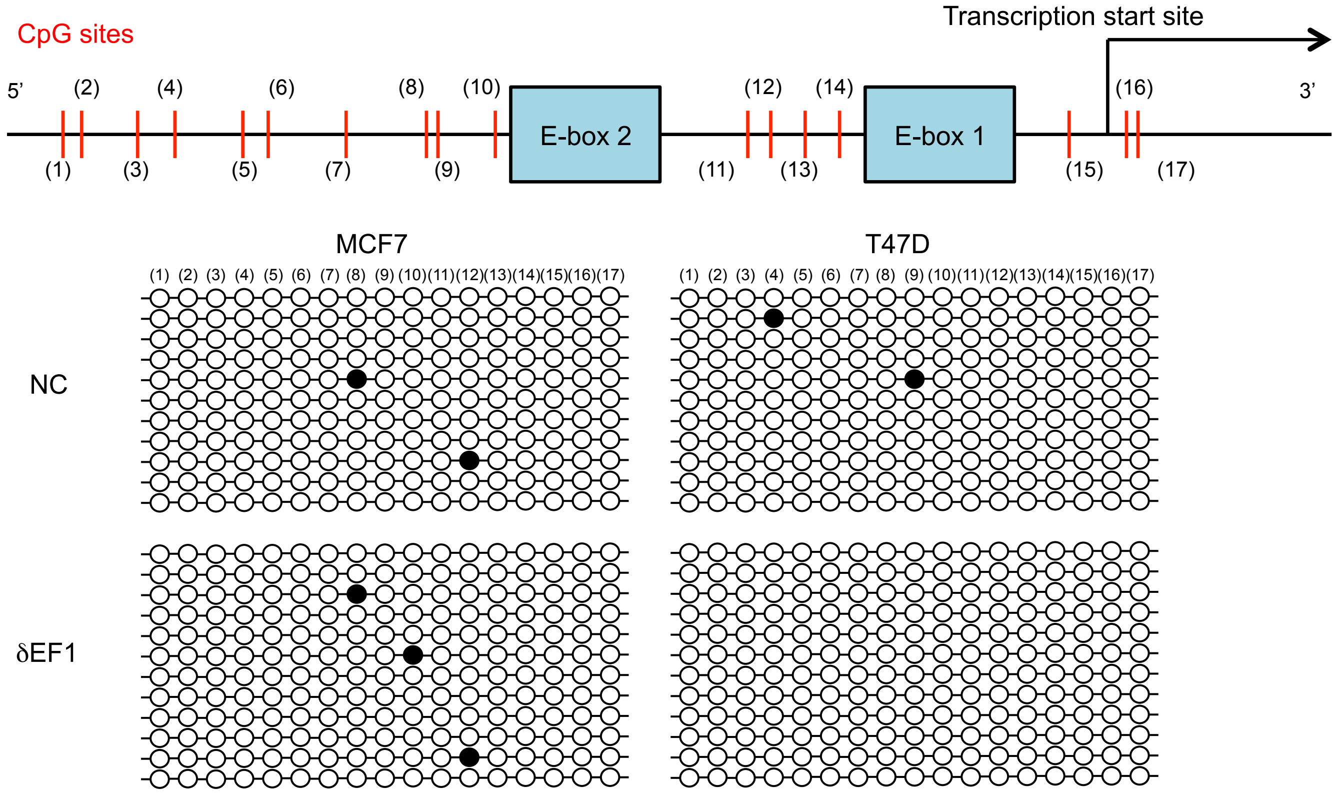

B

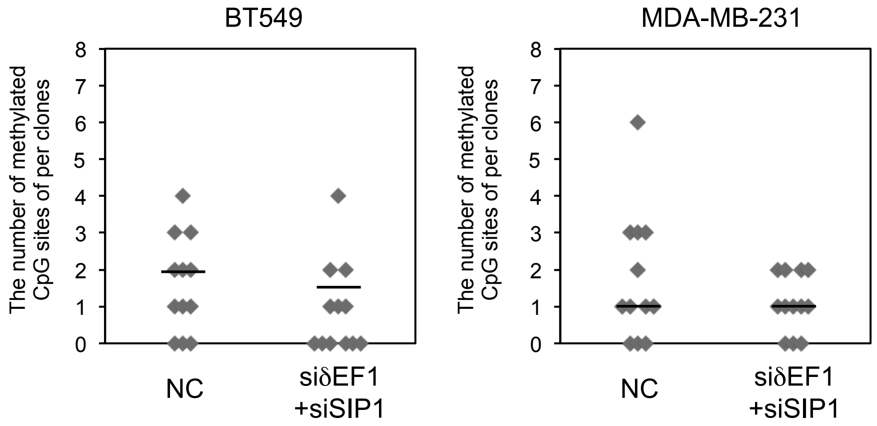

C

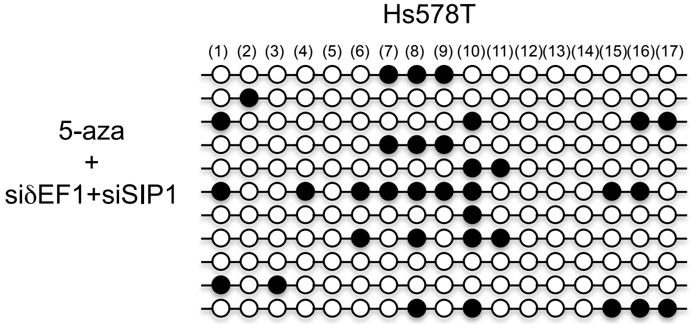

Supplemental figure S2

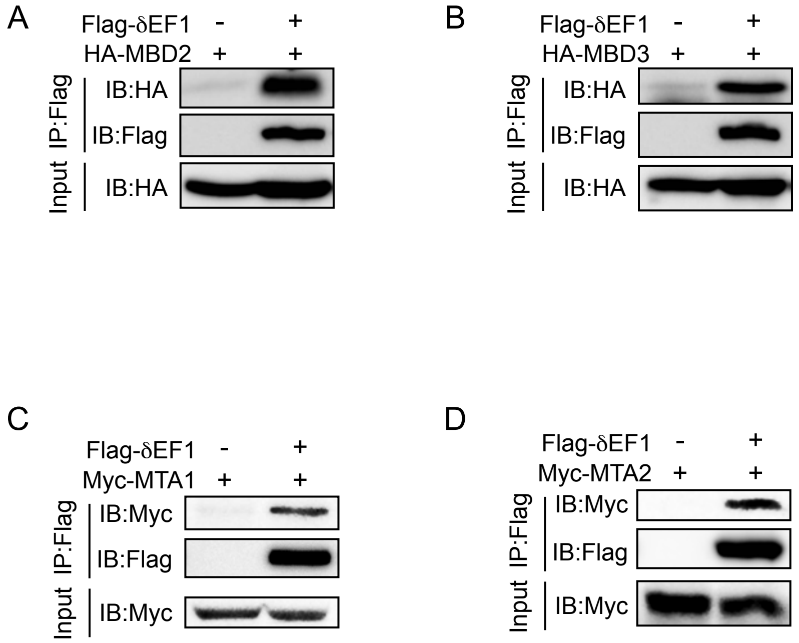

Supplemental figure S3

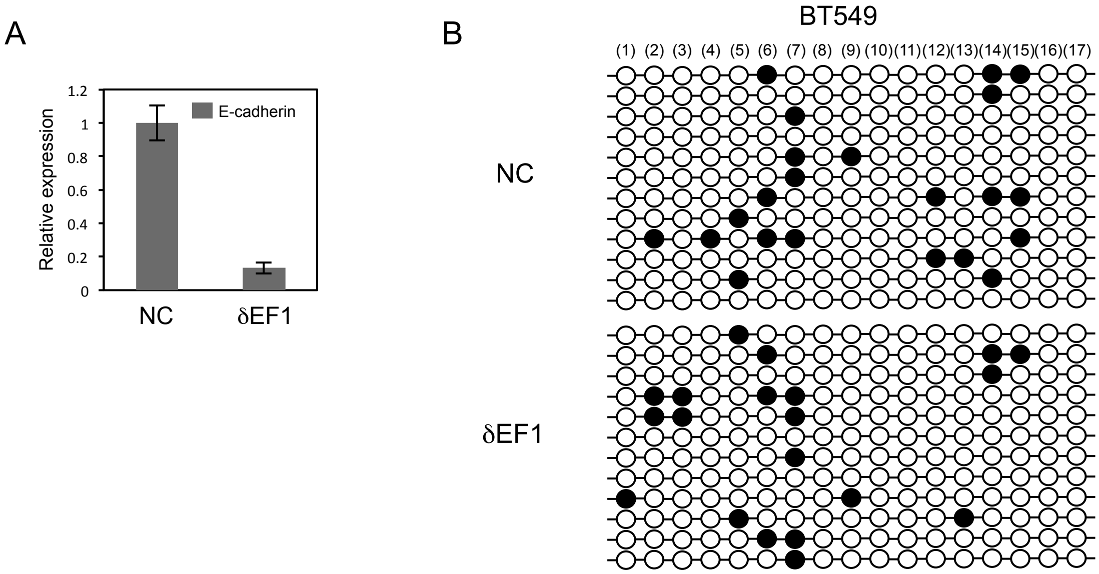

Supplement: Supplementary file 1 [file cam40004-0125-sd1.pdf]
